# Detection of clustered circulating tumour cells in early breast cancer

**DOI:** 10.1038/s41416-021-01327-8

**Published:** 2021-03-24

**Authors:** Ilona Krol, Fabienne D. Schwab, Roberta Carbone, Mathilde Ritter, Sabrina Picocci, Marzia L. De Marni, Grazyna Stepien, Gian M. Franchi, Andrea Zanardi, Marco D. Rissoglio, Alfredo Covelli, Guido Guidi, Daniele Scarinci, Francesc Castro-Giner, Luca Mazzarella, Claudio Doglioni, Francesca Borghi, Paolo Milani, Christian Kurzeder, Walter P. Weber, Nicola Aceto

**Affiliations:** 1 Department of Biomedicine, Cancer Metastasis Laboratory, University of Basel and University Hospital Basel, Basel, Switzerland; 2 Breast Center, University of Basel and University Hospital Basel, 4056 Basel, Switzerland; 3 Tethis S.p.A., Milan, Italy; 4 Genextra S.p.A., Milan, Italy; 5 Swiss Institute of Bioinformatics, Lausanne, Switzerland; 6 Experimental Oncology, IEO European Institute of Oncology IRCCS, Milan, Italy; 7 Department of Pathology, Vita Salute San Raffaele University, IRCCS San Raffaele Scientific Institute, Milan, Italy; 8 CIMAINA and Department of Physics, University of Milan, Milan, Italy; 9 Department of Biology, Institute of Molecular Health Sciences, ETH Zürich, Zürich, Switzerland

**Keywords:** Breast cancer, Cancer screening

## Abstract

Circulating tumour cell (CTC) clusters have been proposed to be major players in the metastatic spread of breast cancer, particularly during advanced disease stages. Yet, it is unclear whether or not they manifest in early breast cancer, as their occurrence in patients with metastasis-free primary disease has not been thoroughly evaluated. In this study, exploiting nanostructured titanium oxide-coated slides for shear-free CTC identification, we detect clustered CTCs in the curative setting of multiple patients with early breast cancer prior to surgical treatment, highlighting their presence already at early disease stages. These results spotlight an important aspect of metastasis biology and the possibility to intervene with anti-cluster therapeutics already during the early manifestation of breast cancer.

## Background

Distant metastasis is accomplished through the hematogenous dissemination of circulating tumour cells (CTCs) from solid primary or metastatic cancerous lesions.^1^ The metastatic cascade is characterised by several rate-limiting steps, including the intravasation of selected tumour cells into the vasculature, their survival within the bloodstream under high-shear conditions, as well as their extravasation and expansion at the target metastatic sites.^2^ The isolation of CTCs from the blood of patients with cancer is enabling a better understanding of the biology of metastasis, alongside with new possibilities for disease monitoring.^3^ In breast cancer, CTCs manifest as single cells or as multicellular aggregates, with the latter featuring higher metastatic ability.^4–7^ This increased metastatic ability of clustered CTCs is attributed to their prominent cell-cell junction repertoire,^4,5,8^ accessibility to binding sites for stemness- and proliferation-associated transcription factors,^6^ metastasis-promoting interactions with white blood cells (WBCs)^7^ or stromal cells,^9^ as well as physical and microenvironment-related properties.^10,11^ However, to date, clusters of CTCs have been observed in patients predominantly during advanced disease stages (i.e. with established distant metastases), raising the question whether or not they also are involved at earlier stages.

## Methods

### Patients

A total of 58 patients, of which 30 were anonymous healthy donors and 28 patients with breast cancer were enrolled in this study. Blood samples were obtained with patient informed consent approved by the institutional review board (protocol EKNZ BASEC 2016-00067, approved by the EKNZ, Ethics Committee northwest/central Switzerland) and conducted according to good clinical practice guidelines. Healthy donor blood was purchased from the Blutspendezentrum SRK beider Basel upon written informed consent of anonymous healthy volunteers. All blood samples were collected through a peripheral, upper extremity vein after discarding the first three millilitres to minimise keratinocytes contamination.

### Sample preparation

Blood samples from patients and healthy donors were collected in EDTA tubes (BD Vacutainer, Beckton Dickinson) and sample preparation for processing was started not later than 45 min after the blood withdraw. Blood samples were processed with gentle red blood cell lysis through ammonium chloride buffer. WBCs were then separated by centrifugation (350 × *g*, 5 min at RT), resuspended in DPBS, counted (Countess II, Life Technologies) and dispensed at a defined concentration on SBS-CTC slides using CellSeed (Tethis S.p.A.). Subsequently, the slides were stored for up 15 days at 4 °C, and then shipped to Tethis laboratory (Milan, Italy) at controlled temperature (4–8 °C).

### CTC Identification

SBS-CTC slides were stained with pan-CK (Sigma-Aldrich, C2562), αER (Cell Signaling, 8644) and CD45-647 (Biorad, MCA87) antibodies. Upon staining, slides were scanned with Metafer platform (ZEISS AxioImagerZ2 microscope). The threshold for single-cell pan-CK positivity of the assay was a signal-to-noise ratio set at the 95th percentile of intensity observed in U937 negative control cells. Similarly, the analytic threshold for single-cell ERα positivity of the assay was a signal-to-noise ratio set at the 95th percentile of intensity observed in U937 negative control cells. HER2 gene amplification was detected by FISH analysis (performed after immunofluorescence staining with Pan-CK, CD45 and ER) using HER2 probes (ERBB2 (HER2/NEU) amp probe, Metasystems). The operator was blinded in regard to sample identity.

### CTC capture rate

A defined number of BR16-RFP single cells and clusters (human CTC-derived cell line) was spiked into healthy donor blood (containing 5 × 10^6^ WBCs), incubated at RT for 15 min and processed as described above (shortly: RBCs lysis was followed by WBCs counting, mixing with BR16 cells and loading into CellSeed). After automated cell distribution, BR16-RFP cells on SBS-CTC slides were counted using Leica (DMIL) microscope. The data were collected from three independent experiments.

### Copy number analysis

The sequencing reads were assessed for quality control using FastQC v.0.11.4 (http://www.bioinformatics.babraham.ac.uk/projects/fastqc/) and trimmed using Trim_Galore. Trimmed reads were aligned to human reference genome (GRCh38) using bowtie2 v2.3.4.2 and retaining only reads with a mapping quality above 20. Filtered reads were sorted using Samtools v1.7 and duplicates removed using Picard v2.9.2 (http://broadinstitute.github.io/picard/). Resulting BAM files were converted to BED files using the bamToBed from BEDtools v2.27.1(https://bedtools.readthedocs.io/en/latest/). CNV analysis was performed with Ginkgo and the following parameters: variable bins of 500 kb size and segmentation using normalised read counts.

## Results

**Fig. 1:**
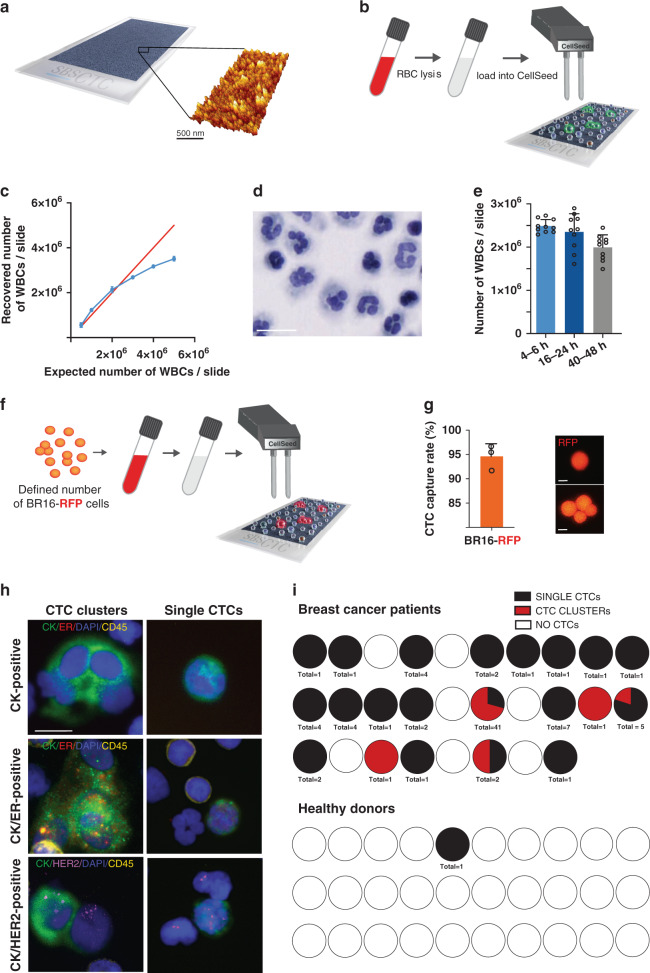
Setup of SBS-CTC technology for shear-free CTC capture. **a** Graphical representation of the SBS surface and its corresponding 3D structure. **b** Schematic representation of CTC capture strategy with the CellSeed device. CTCs and WBCs are seeded and captured on the SBS-CTC slides after RBCs depletion. **c** WBC adhesion efficiency curve on SBS-CTC slide; the recovered number of WBC/slide has been plotted versus the expected number of WBC/slide for six WBC concentrations (*n* = 3). Error bars represent SD. The linear regression was calculated: *y* *=* *−1E−07x*^*2*^ *+* *1.2441x−28887, R*^*2*^ *=* *0.999*. **d** Representative image of Papanicolaou-stained WBCs and corresponding cell morphology and distribution (scale bar 20 μm). **e** Influence of sample processing time on WBCs adhesion efficiency. Sample processing time points have been set at 4–6 h, 16–24 h and 40–48 h upon the blood draw. Box plots showing mean value of WBCs adhered to SBS (*n* = 10 for all, 5 slides/each sample). Error bars represent SD. **f** Graphical representation of the procedure to assess CTC capture rate on SBS-CTC slides. A defined number of BR16-RFP single and clustered CTCs were spiked into healthy donor blood and processed with CellSeed to assess on-slide capture rate. **g** Plot showing the capture efficiency of the SBS-CTC surface using CellSeed device for BR16-RFP cells spiked in healthy donor blood samples (left; error bars represent SD). Representative images of captured BR16-RFP single and clustered cells are shown (right; scale bar 10 μm). **h** Representative immunofluorescence images of captured CTC clusters (top) and single CTCs (bottom) stained for pan-cytokeratin (CK, green), oestrogen receptor α (ER, red) and DAPI (nuclei, blue). The same cells were also interrogated for ErbB2 gene amplification by fluorescence in situ hybridisation (HER2, magenta). White blood cells were stained for CD45 (yellow). Scale bar (10 μm). **i** Pie charts showing the raw distribution of detected single CTCs (black) and CTC clusters (red) in each analysed blood sample. The total number of CTCs is shown (bottom).

Capture of clustered CTCs from the blood of cancer patients is a challenging task. Not only are CTC clusters highly diluted in the peripheral circulation of patients,^7^ but they may also be subject to disaggregation during processing with microfluidic devices due to constant shear stress—typically exploited by many CTC enrichment procedures. To this purpose, we have focused our efforts on setting up a new method for CTC identification that could fully preserve both biological and phenotypic integrity of clustered cells in a given blood sample, purposely designed to avoid shear stress. We made use of Smart BioSurface slides (SBS-CTC)—characterised by a biocompatible surface composed of nanostructured titanium oxide—capable to spontaneously immobilise all cell types with high efficiency, at room temperature and in a shear-free fashion (Fig. 1a), thanks to the formation of integrin-based focal adhesion points with living cells.^12,13^ To achieve standardisation of slide preparation, we made use of a customised liquid handling platform, CellSeed, for automated cell seeding and fixation directly on SBS-CTC slides. With these tools, upon red blood cell (RBC) lysis, unmanipulated blood specimens are uniformly seeded on SBS-CTC slides in the absence of shear stress, i.e. converting a liquid biopsy into a two-dimensional tissue slide alongside with full maintenance of the morphological features of its cellular components (Fig. 1b). Bearing in mind future translation into clinical practice and therefore desired standardisation of sample handing, we have customised the CellSeed platform in a fully automated way (from cell seeding to fixing and washing; see ‘Methods’). This provides efficiency to the process, resulting in the ability to achieve seeding of up to 20 slides (corresponding to ca. 12 ml of blood) in 2 h. We further optimised our strategy to make sure that no artificial (on-slide) clustering of cells would occur during seeding, yet preserving the homogeneity of cell distribution and the ability to process a high number of cells per slide. To this end, we obtained healthy donor (HD) blood samples and upon RBC lysis, different concentrations of WBCs—from 0.5 × 10^6^ to 5 × 10^6^ cells per slide—were seeded and fixed with CellSeed, stained with DAPI and analysed by automated cell count. We found the optimal concentration to be at 2.5 × 10^6^ WBCs per slide, i.e. where the polynomial curve representing the experimental adhesion efficiency on SBS-CTC slides intercepts the linear equation, indicating maximum adhesion efficiency while avoiding the formation of artificial clusters due to cell overcrowding (Fig. 1c). With Papanicolaou (PAP) staining, we also confirmed excellent sample integrity, as suggested by intact nuclei and cytoplasm of blood cells (Fig. 1d). Further, when testing primary blood collection methods, we found that EDTA tubes were optimal to avoid artificial blood cell clustering, as opposed to Cell Free DNA BCT tubes that positively interfere with cellular aggregation (Supplementary Fig. 1). We also found that the time that passes from blood draw until its processing is a key parameter influencing cell adhesion to the SBS-CTC slides. Particularly, we found that adhesion is optimal (99.1%) for samples processed within 4–6 h, while it decreases for longer incubation times (Fig. 1e). With these optimised processing parameters, we assessed CTC capture rate on SBS-CTC slides by spiking a defined number (ranging from 66 to 91) of RFP-labelled single and clustered CTCs from the BR16 model—a human CTC-derived cell line^6^—in healthy donor blood, and processed the samples as described above (Fig. 1f). We confirmed a mean CTC capture efficiency of 94.6%, along with complete maintenance of morphological features of both single and clustered cells (Fig. 1g). Alongside, we confirmed no artificial cluster formation during processing and high reproducibility of cell recovery rates (Supplementary Figs. 2, 3). Together, we established a new technology, SBS-CTC slides coupled with CellSeed device, capable to transform a liquid biopsy into a two-dimensional tissue slide, achieving highly efficient CTC capture and preservation of morphological features of cells, thus enabling subsequent non-disruptive CTC analysis in clinical samples.

The establishment of the SBS-CTC technology in combination with CellSeed device allowed us to proceed with shear stress-free interrogation of clinical samples, aiming at the assessment of physiological CTC composition in patients with early breast cancer. To this end, we enrolled a cohort of 58 patients, of which 30 were anonymous healthy donors and 28 were patients with early breast cancer (i.e. without distant metastasis) prior to surgical treatment. The mean age of the breast cancer patients was 67 years (range 33–87). We included 11 patients with AJCC stage IA, nine patients with stage IIA, four patients with stage IIB, two patients with stage IIIA, and two patients with axillary relapse. No patient with stage IV disease was included. Neoadjuvant chemotherapy was given to 5/28 breast cancer patients, while 2/28 patients had neoadjuvant letrozole. Tumour subtype was luminal-like in 21 patients,^14^ triple-negative in four patients and HER2-positive in three (of which two were hormone receptor negative and one was hormone receptor positive, respectively) (Supplementary Table 1). In all patients, blood was withdrawn in EDTA tubes before surgery, and the first tube was discarded to avoid keratinocyte contamination. For CTC enumeration purposes, we considered as CTCs those cells presenting with cytokeratin (CK)-positivity, CD45-negativity and cancer-related morphological features (e.g. large nucleolus, irregular size and shape, scarce cytoplasm), plus at least one of the following parameters: presence of multicellular aggregates of CK-positive cells (at least two cells), positivity for the oestrogen receptor (ER) or gene amplification of the tyrosine kinase receptor ErbB2 (HER2, evaluated by fluorescence in situ hybridisation). Strikingly, we could detect clustered CTCs (both cancer cells only and cancer cells clustered with immune cells) in five patients with early breast cancer, namely three with stage IA and two with stage IIA (Fig. 1h and Supplementary Table 1). The three patients with stage IA were all post-menopausal women; one of them had a luminal-A like tumour and two had luminal-B like tumours. The two patients with stage IIA disease were also post-menopausal women with a luminal-B like tumour and a HER2-positive, ER/PR-negative tumour, respectively. Of note, we could detect most CTC clusters (29 clusters containing a total of 236 cancer cells in a volume of 5.05 ml of blood) in the blood of a patient diagnosed with luminal-A like disease (Supplementary Fig. 4). Additionally to the five patients that resulted positive for CTC clusters, we found single CTCs in 16 patients, while we could not detect any single or clustered CTC in seven breast cancer patients (two stage IA patients, four stage IIA patients, and one patient with stage IIB, respectively) (Fig. 1i, S4 and Supplementary Table 1). More generally, we also observed a high concordance between ER and/or HER2 positivity in CTCs and their status in the primary tumour, namely 94.1% concordance for ER-positive and 100% concordance for HER2-positive cases (Supplementary Fig. 5). As control, the vast majority (29/30) of healthy donors resulted negative for both single CTCs and CTC clusters, as expected, with the exception of one donor in whom one single CTC was found (Fig. 1i, S4 and Supplementary Table 1). Finally, we confirmed neoplastic identity of isolated CTC clusters by means of copy number variation (CNV) analysis of multiple individual clusters isolated from the same patient, alongside with WBC controls (Supplementary Fig. 6). Together, interrogation of blood samples with SBS-CTC technology not only revealed the presence of CTC clusters in early breast cancer, but also enabled CTC detection in a high proportion of breast cancer patients at 75% sensitivity and 96.7% specificity when considering all subtypes, or 81% sensitivity when considering the luminal-like subtypes (Supplementary Table 2).

These results provide proof-of-concept evidence of the presence of CTC clusters in early breast cancer, suggesting their possible involvement in early metastatic spread and disease progression. To evaluate whether or not CTC clusters are present in patients with localised disease we made use of a non-disruptive technology that allows CTC visualisation in a shear-free fashion, enabling morphological preservation of all cells and avoiding shear-based disruption of multicellular aggregates. We identify CTC clusters of small and large size (from two to more than 50 cells) in patients with luminal-A-like (*n* = 1/8), luminal-B-like (*n* = 3/13) or HER2-positive (*n* = 1/3) disease, while no CTC clusters were found in patients with triple-negative breast cancer (*n* = 0/4). These results reveal the involvement of CTC clusters very early on upon breast cancer diagnosis, highlighting a previously-unappreciated feature of CTC and metastasis biology. However, given the very early stage of disease for patients in our cohort, disease progression was not yet observed. While CTC clusters biology has been subject to recent investigations that revealed their metastatic propensity,^4,6–8^ the underlying causes for CTC cluster generation from a cancerous lesion are understood only in part, with intra-tumour hypoxia being recently implicated.^11^ Similarly to single CTCs, however, it is difficult to predict which patients will be positive for CTC clusters, yet their presence has been linked to a poor prognosis in several independent studies.^4,7,15–19^

## Conclusions

Identification of CTC clusters in early breast cancer has several implications. First, it excludes that CTC clusters are an exclusive feature of metastatic disease, suggesting that they may be a general phenomenon of breast cancer biology independently of disease stage. Second, clinical trials are ongoing to evaluate the effects of cluster-targeting agents in the metastatic setting (e.g. NCT03928210), and the possibility to apply these agents early during disease progression may decrease the risk to develop distant metastasis. Third, the presence of CTC clusters in early breast cancer may be considered as a further, substantial risk factor for disease progression, yet larger clinical trials will be needed to address this point. These trials should include patients with different tumour subtypes and stages, along with CTC-isolation technologies that are designed to avoid disruptive elements that could damage multicellular aggregates and favour the identification of single cells, such as high shear stress, filtering and high flow velocity.

Altogether, our results expand the current knowledge on CTC clusters as major players in the metastatic process of breast cancer. Using SBS-CTC technology, we observe them in the peripheral circulation of patients with non-metastatic early disease, shortly after diagnosis and prior to surgical intervention in curative intent, highlighting the need for larger prospective studies to address their prognostic potential in various breast cancer subtypes and eventually other cancer types, possibly including staining with additional markers to further improve sensitivity. While our study has clearly a proof-of-concept character with a rather limited and heterogeneous cohort of patients, with cluster-targeting therapies being developed, the identification of patients with CTC clusters may help to pinpoint high-risk individuals and to render them eligible for anti-cluster treatments very early on, aiming to reduce the metastatic ability of cancer cells.

## Supplementary information

Supplementary Information

## Data Availability

The supporting data used in this manuscript can be requested and will be provided by the corresponding author, Prof. Nicola Aceto.
