# Supplementary material for: Detection of clustered circulating tumour cells in early breast cancer
Source: Br J Cancer. 2021 Mar 24;125(1):23–7. doi: 10.1038/s41416-021-01327-8 (PMC8257701; doi:10.1038/s41416-021-01327-8)
Supplement: Supplementary file 1 — Supplementary Information [file 41416_2021_1327_MOESM1_ESM.pdf]

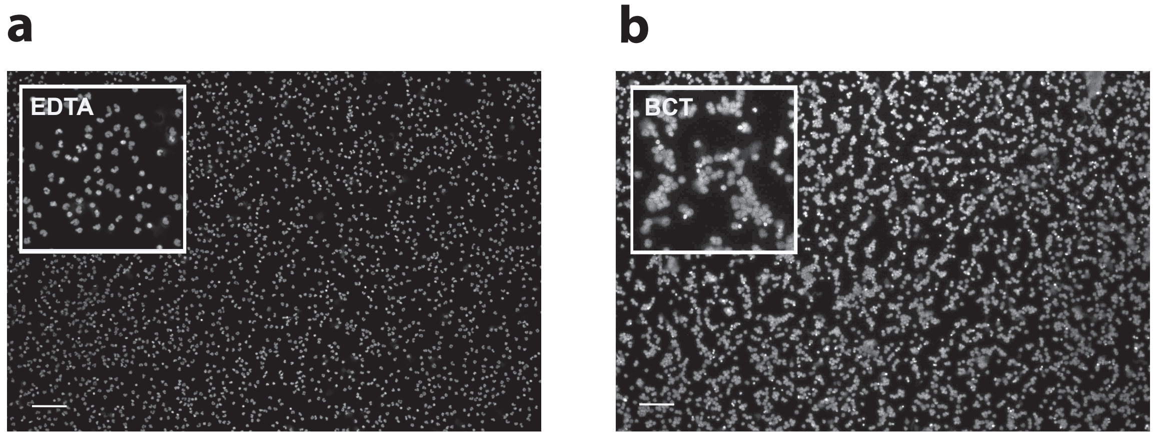

Supplementary Figure 1. Effects of blood preservative on white blood cell (WBC) adhesion homogeneity on SBS-CTC slides. (**a**) Representative image of WBC distribution within a SBS-CTC slide for a sample collected in EDTA tubes (scale bar 100μm). We observe no evidence of artificial cluster formation. (**b**) Representative image of WBC distribution within a SBS-CTC slide for a sample collected in BCT tubes (scale bar 100μm). In this case, artificial cluster formation is observed.

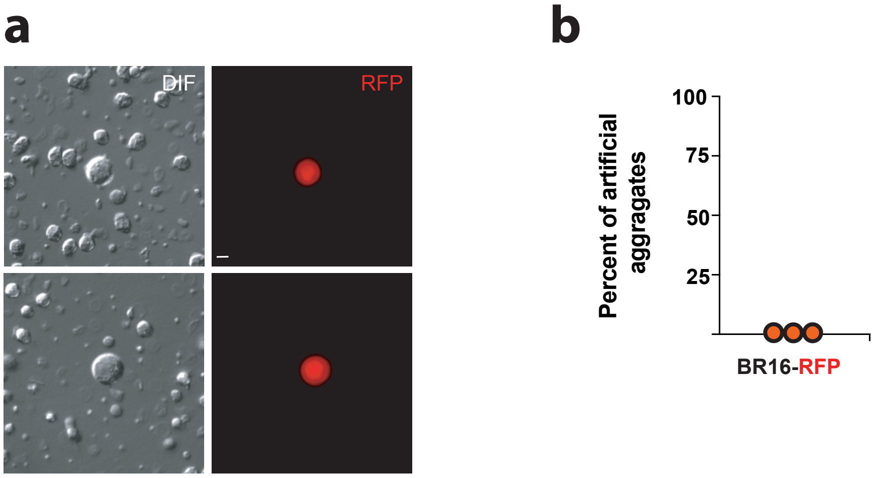

Supplementary Figure 2. No evidence of artifcial cluster formation. (**a**) Representative images of single BR16-RFP cells captured on the SBS-CTC slide (scale bar 10µm). (**b**) Plot showing percent of artificial aggregates on the SBS-CTC surface upon spiking 50 single BR16-RFP cells in healthy donor blood samples.

Suppl. Figure 2

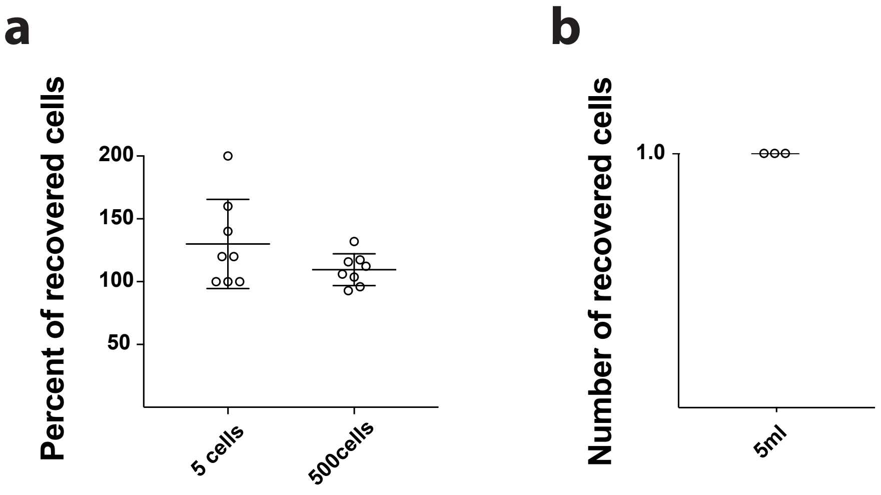

Supplementary Figure 3. Reproducibility of the results. (a) The plot shows the percent of recovered cells in multiple instances (n=8) using 5 or 500 BR16-RFP cells spiked in healthy donor blood per each SBS-CTC slide. Error bars represent SD. (b) The plot shows the number of recovered cells when spiking 1 BR16-RFP cell in 5ml of healthy donor blood (n=3).

Suppl. Figure 3

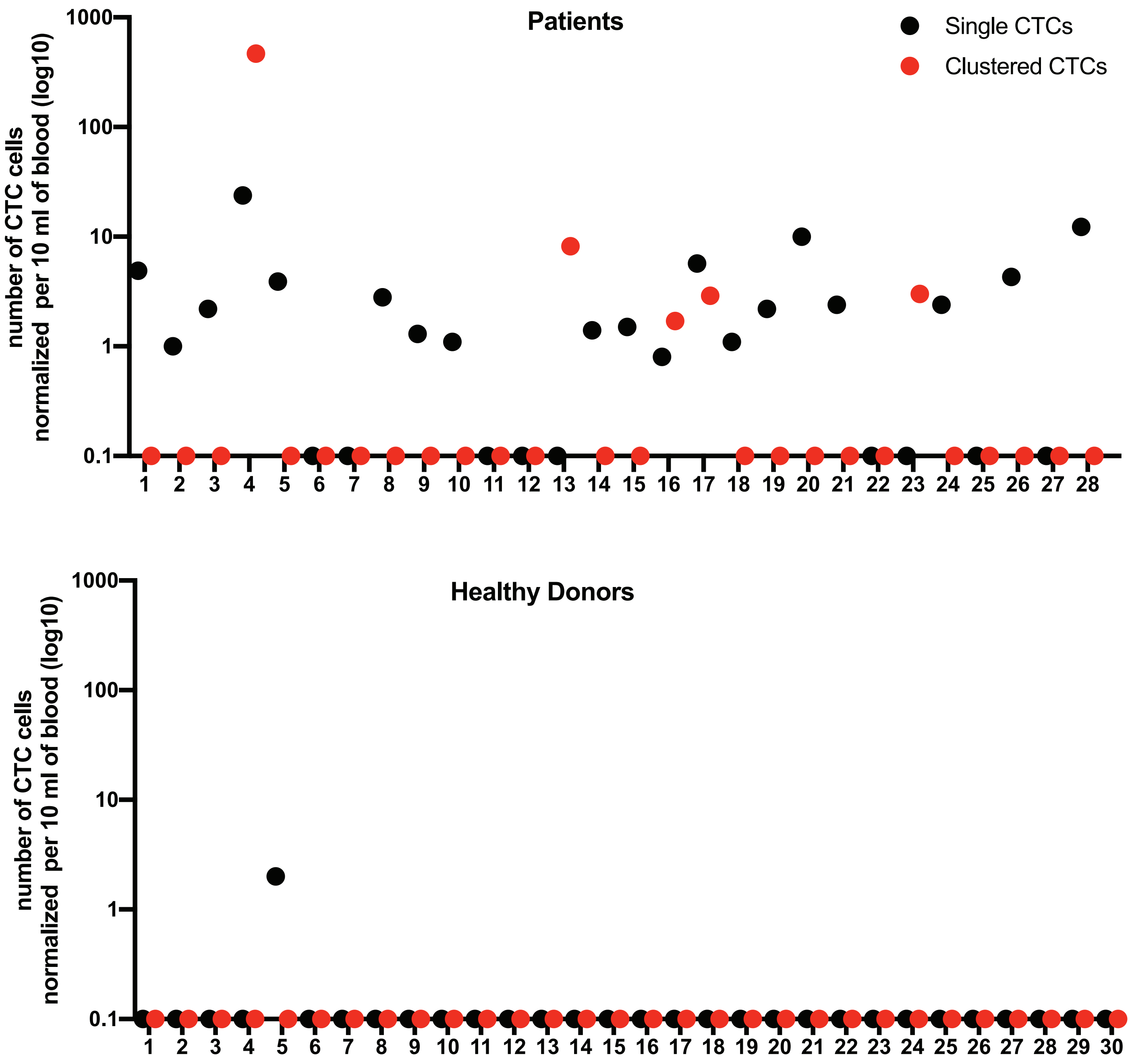

Supplementary Figure 4. Number of detected CTCs in each sample. The plots show normalized (per 10 ml blood volume) number of detected single (*black*) and clustered (*red*) circulating tumour cells in breast cancer patients (*top*) and healthy donors (*bottom*). Values for clustered CTCs are displayed considering the number of cells in clusters.

Suppl. Figure 4

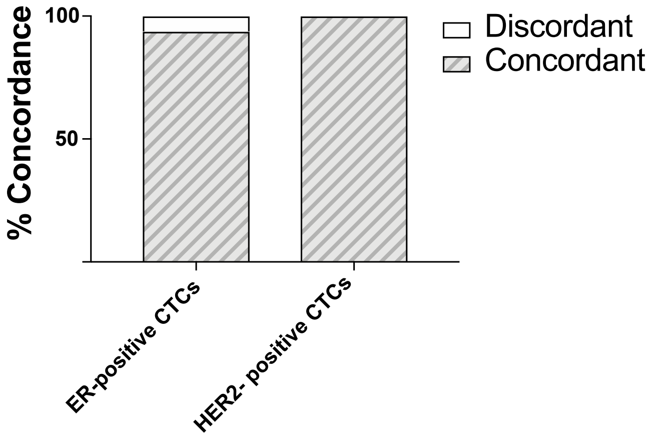

Supplementary Figure 5. Concordance between CTC marker positivity and primary tumour. The plot shows the percent of CTCs in which marker status (ER and HER2) is concordant with that of the primary tumour.

Suppl. Figure 5

White blood Cells

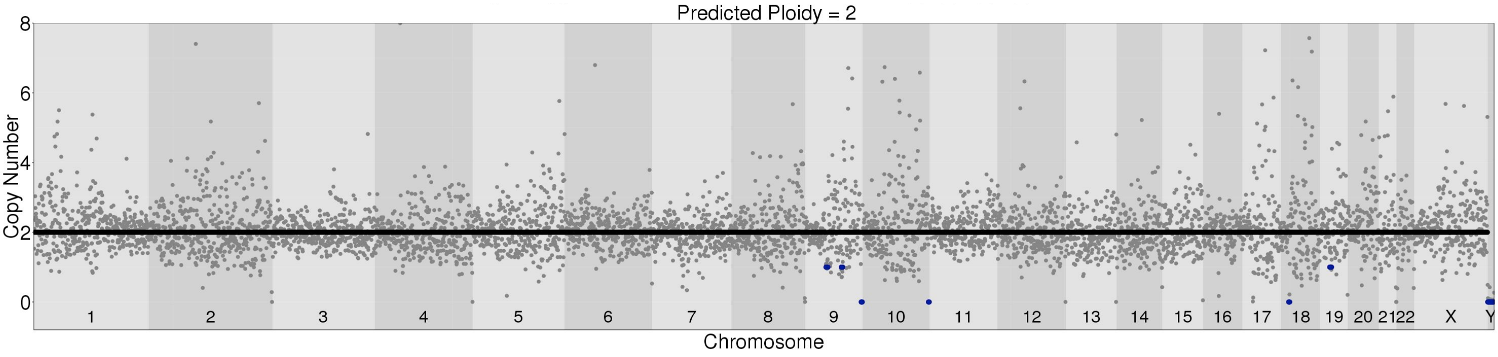

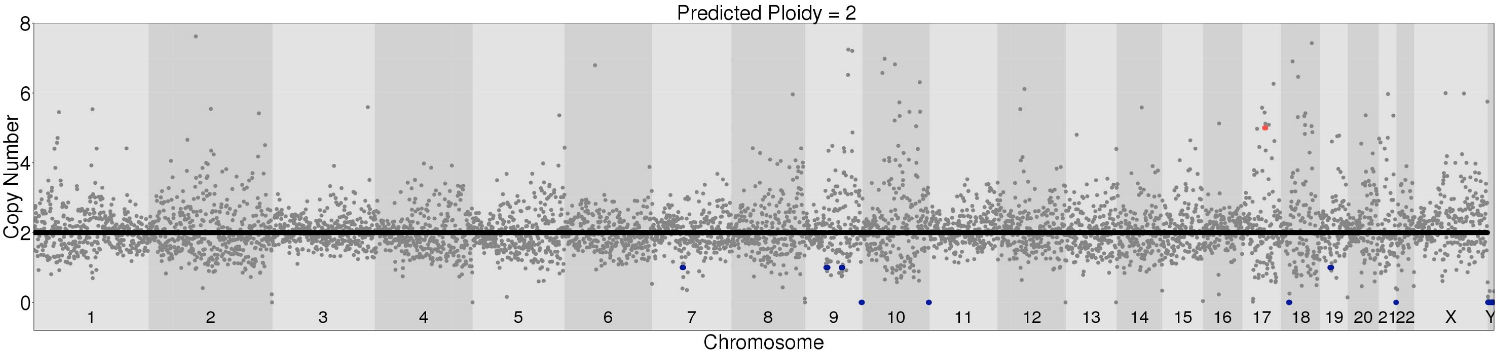

CTC clusters

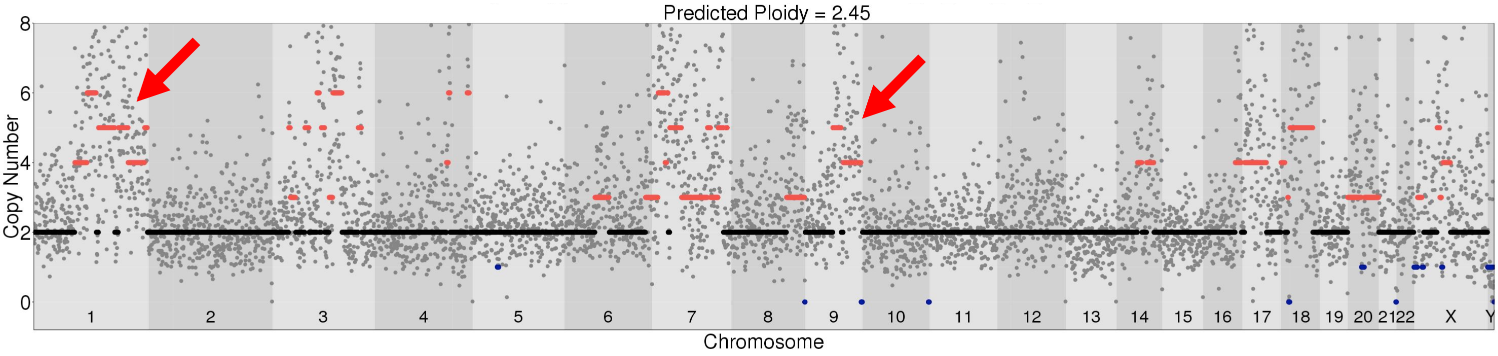

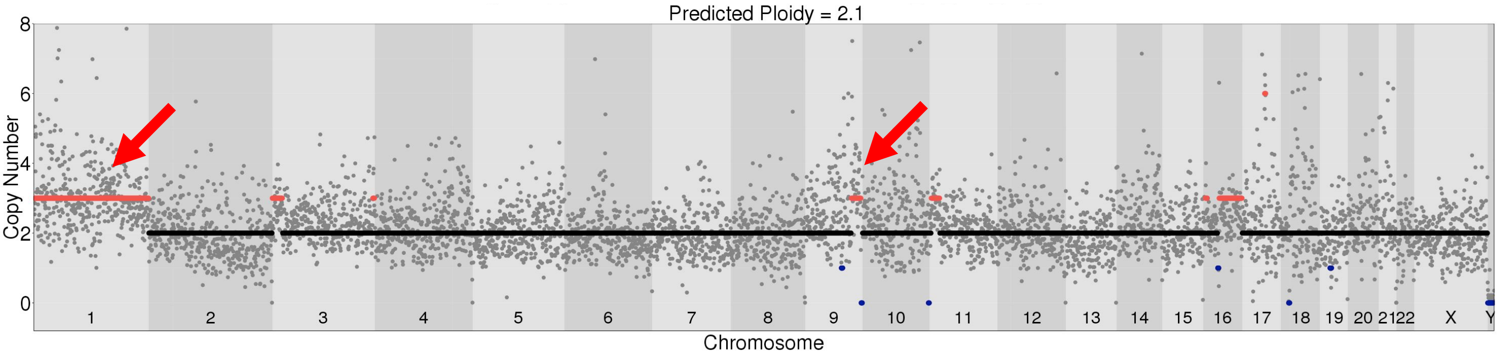

Supplementary Figure 6. Copy number profiles of white blood cell controls and CTC clusters. In particular, both CTC clusters are isolated from the same patient (ID #4), and display commonalities in the copy number profile, i.e. amplification of parts of Chromosome 1 and 9 (red arrows), alongside with additional changes that are specific to each cluster. In contrast, white blood cells present a normal (diploid) copy number profile.

Suppl. Figure 6

Pat. ID	Age	Meno-pausal status	ER	PR	HER2	Ki-67	Initial AJCC Stage 7th edition	Subtype	Neo-adjuvant treatment	Pre-Op. TNM	Postoperative TNM	pCR	Number of CK-positive CTC clusters	Number of CK-ER-positive CTC clusters	Number of CK-positive HER2-amplified CTC clusters	Number of CK-ER-positive single CTCs	Number of CK-positive HER2-amplified single CTCs
1	76	post	100%	80%	neg.	5%	IA	lumA	no	-	pT1c pN0 (0/1)cM0 G1 V0 L0 Pn0 R0	-	0	0	0	4	0
2	80	post	100%	100%	neg.	5%	IA	lumA	no	-	pT1c pN0 (0/1)cM0 G2 V0 Pn0 L1 R0	-	0	0	0	1	0
3	83	post	95%	80%	neg.	15%	IA	lumA	no	-	pT1b pN0 (0/1)cM0 G1 L0 V0 Pn0 R0	-	0	0	0	2	0
4	55	post	100%	80%	neg.	10%	IA	lumA	no	-	pT1c pN0 (0/2)cM0 G1 V0 L0 Pn1 R0	-	4 cluster (2cells), 9cluster (3cells), 4cluster (4cells), 3cluster (5cells), 4cluster (6cells), 1cluster (13cells), 1cluster (15cells), 1cluster (33cells), 1cluster (35cells), 1cluster (>50cells)	0	0	12	0
5	76	post	100%	100%	neg.	5-10%	IA	lumA	no	-	pT1c pN0 (0/3)cM0 G1 L0 V0 Pn0 R0	-	0	0	0	2	0
6	56	post	90%	100%	neg.	5%	IIA	lumA	no	-	pT2 pN0 (0/2)cM0 G1 V0 L0 Pn0 R0	-	0	0	0	0	0
7	46	pre	90%	90%	neg.	5-10%	IIA	lumA	no	-	pT1c pN1 (3/11)iM0 G2 V0 L1 Pn1 R0	-	0	0	0	0	0
8	83	post	80%	80%	neg.	5%	IIA	lumA	Letrozol for 2 month	cT3c N0c M0	ypT2 ypN0 (0/3)cM0 G1 V0 L0 Pn0 R0	no	0	0	0	1	0
9	71	post	100%	90%	neg.	20%	IIIA	lumB	no	-	rpT1c pN2a (4/17)iM0 G2 L0 V0 Pn0 R0	-	0	0	0	0	1
10	85	post	60%	50%	neg.	25%	IIB	lumB	Letrozol for 20 months	cT2c Noc M0	ypT2a pN1mi(1/3)iM0 G2 V0 Pn0 L1 R0	no	0	0	0	1	0
11	65	post	95%	30-40%	neg.	70%	IIB	lumB	Epirubicin Cyclo-phosphamid Taxol	cT2c N1c M0	ypTis ypN0 (0/4)cM0 G3 V0 L0 Pn0 R0	no, residual DCIS, no cancer (Regression after Sinn:	0	0	0	0	0

												3)					
12	87	post	90%	10%	neg.	30%	IIA	lumB	no	-	pT2 cN0 cM0 G2 L0 V0Pn0 R0	-	0	0	0	0	0
13	84	post	90%	10%	neg.	20%	IA	lumB	no	-	pT1a pN0 (0/1)cM0 G2 V0 L1 Pn0 R0	-	0	1 cluster (4cells)	0	0	0
14	74	post	100%	10%	neg.	40%	IIA	lumB	no	-	pT2 pN0 (0/6)cM0 G3 L1 V0 Pn1 R0	-	0	0	0	1	0
15	54	post	100%	10%	neg.	15%	IIA	lumB	no	-	pT1c pN1a(1/16)iM0 G2 L1 V0 Pn0 R0	-	0	0	0	1	0
16	73	post	100%	90%	neg.	35%	IIA	lumB	no	-	pT2(m) pN0 (0/3)cM0 G2 L1 V0 Pn1 R0	-	1 cluster (2cells)	0	0	1	0
17	63	post	100%	20%	neg.	20%	IA	lumB	no	-	pT1c pN1mi (1/2)cM0 G1V0 L0 Pn0 R0		1 cluster (2cells)	0	0	4	0
18	81	post	100%	1%	neg.	10%	IA	lumB	no	-	pT1 pN0 (0/1)cM0 L0 V0 R0	-	0	0	0	1	0
19	33	pre	95%	95%	neg.	20%	IIIA	lumB	no	-	pT3 pN2a (8/22)iM0 G2 L1 V0 Pn0 R0	-	0	0	0	1	0
20	54	post	90%	5%	neg.	30%	IA	lumB	no	-	pT1b pN0 (0/1)cM0 G2 V0 L0 Pn0 R0	-	0	0	0	4	0
21	50	pre	50%	60%	neg.	60%	recurrent tumor	lumB	no	-	rpN1a(2/2) Pn1	-	0	0	0	1	0
22	80	post	90%	5%	pos.	20%	IA	HER2	no	-	pT1b pN0 (0/2)cM0 G2 V0 Pn0 L0 R0	-	0	0	0	0	0
23	55	post	0%	0%	pos.	70%	IIA	HER2	Docetaxel Carboplatin Herceptin Pertuzumab	cT2c N0c M0	ypT1a ypN0 (0/6)iM0 G3 L0 V0 pN0 R0	no	0	0	1 cluster (2 cells)	0	0
24	85	post	0%	0%	pos.	25%	IIB	HER2	Taxol Herceptin Pertuzumab	cT2c N1c M0	ypT0 ypN0 (0/6)cM0 L0 V0 Pn0 R0	yes	0	0	0	1	1
25	66	post	0%	0%	neg.	50%	IA	Triple neg.	no	-	pT1c pN1a (2/3)iM0 G3 L0 V0 Pn0 R0	-	0	0	0	0	0
26	42	pre	0%	0%	neg.	75%	IIB	Triple neg.	Adriblastin Cyclo-phosphamid Paclitaxel Carboplatin	cT2c N1c M0	ypT0 ypN0 (0/4)iM0 G3 V0 L0 Pn0 R0	yes	0	0	0	4	0
27	78	post	0%	0%	neg.	50%	IIA	Triple neg.	Carboplatin Paclitaxel	cT2c N0c M0	ypT0 ypN0(0/1)iM0 G3 L0 V0 Pn0 R0	yes	0	0	0	0	0
28	36	post (GnRH, Chemo)	0%	0%	neg.	not available	axillary Re-currence	Triple neg.	no	-	rpN2a (8/16)cM0 G3	-	0	0	0	7	0

Supplementary Table 1. Clinical features of enrolled patients and features of detected CTCs. For each patient, the table shows patient identification number (ID), age, menopausal status, ER, PR, HER2 and Ki-67 status of the primary tumor, initial AJCC Stage, Subtype: lumA= luminal A-like, lumB= luminal-B like, HER2, triple negative, whether and how the patient was treated neoadjuvantly, pre-and post TNM, Abbreviation: L=Lymphatic invasion, V= Vascular invasion, Pn=Perineural invasion, pCR, Number of detected pan-cytokeratin (CK)-positive CTC clusters, the number of detected CK- and estrogen receptor (ER)-positive CTC clusters, the number of detected CK-positive and HER2-amplified CTC clusters, the number of detected CK- and ER-positive single CTCs and the number of detected CK- and HER2-amplified single CTCs.

Disease Condition	n	≥1 CTC detected by SBS: n (%)
**Breast Cancer Patients Cohort**	**28**	**21 (75)**
Luminal-like Breast cancer patients	21	17 (81)
**Healthy Donors Cohort**	**30**	**1 (3.3)**

Supplementary Table 2. **CTC detection rates in breast cancer patients and healthy donors**. The table shows disease condition, number of samples (n) and number of patients in whom at least 1 CTC was detected with the SBS-CTC technology. The corresponding percentage of patients that resulted CTC-positive is shown in brackets.
